# Antibacterial Effects and Mechanism of Mandarin (*Citrus reticulata* L.) Essential Oil against *Staphylococcus aureus*

**DOI:** 10.3390/molecules25214956

**Published:** 2020-10-26

**Authors:** Xueying Song, Ting Liu, Lei Wang, Liu Liu, Xiaoping Li, Xiaoxia Wu

**Affiliations:** College of Food Engineering and Nutrition Science, Shaanxi Normal University, Xi’an 710119, Shaanxi, China; sxy18734445184@163.com (X.S.); Ting_liu@126.com (T.L.); wanglei19950707@163.com (L.W.); xiaoxiaw@snnu.edu.cn (X.W.)

**Keywords:** citrus essential oil, *Staphylococcus aureus*, cell membrane, food safety

## Abstract

*Staphylococcus aureus* (*S. aureus*) creates an array of challenges for the food industry and causes foodborne diseases in people, largely due to its strong antibiotic resistance. Mandarin (*Citrus reticulata* L.) essential oil (MEO) is recognized as a natural and safe preservative; however, the antibacterial effects and mechanism of MEO to combat *S. aureus* are not yet clearly understood. This study will examine the inhibitory effects of MEO against *S. aureus* and explore the antibacterial mechanism thereof from the perspective of membrane destruction. The antibacterial activity of MEO on planktonic *S. aureus* was examined to determine the minimal inhibitory concentration (MIC). Scanning electron microscope (SEM) images revealed the direct impacts of MEO treatment on the cell structure of *S. aureus*. The cell membrane was observed to be depolarized, the determination of extracellular nucleic acids, proteins and intracellular adenosine triphosphate (ATP) confirmed the increased permeability of the cell membrane, its integrity was destroyed and the cellular constituents had leaked. These results, thus, provided conclusive evidence that MEO constrains the growth of planktonic *S. aureus* by affecting the permeability and integrity of its cell membrane. Our study provides a basis for the further development and utilization of MEO as a natural antibacterial agent in the food industry.

## 1. Introduction

Citrus are widely popular all over the world. The *Citrus* genus belongs to the Rutaceae family and is characterized by unique aromas and pleasant tastes [1]. Common varieties of citrus include oranges, limes, lemons and grapefruits [2]. Essential oils (EOs) act as natural antimicrobial agents. They are extracted from a variety of aromatic herbs and fruits, and are gradually increasing in popularity as safe, natural alternatives to artificial chemical compounds in the food industry, due to greater consumer acceptability. The extensive biological activities of EOs have been widely used in food chemistry, pharmacology, pharmaceutics and related fields [3]. Numerous previous studies have described their antioxidant, antiradical and antimicrobial effects [4,5,6]. Citrus essential oils are well known for their flavor and fragrance properties, as well as numerous aromatherapeutic and medicinal applications [7]. The nanoemulsion of EO from *Citrus medica* L. var. *sarcodactylis*, for example, has been reported to exhibit significant antioxidant, antibacterial and antibiofilm activities [8], as well as an inhibitory effect on bacteria in tofu. To compared the inhibitory activity of different citrus essential oils (lemon (*Citrus lemon* L.), mandarin (*Citrus reticulata* L.), grapefruit (*Citrus paradisi* L.) and orange (*Citrus sinensis* L.) against several fungi (*Aspergillus niger*, *Aspergillus flavus*, *Penicillium chrysogenum* and *Penicillium verrucosum*), and found that mandarin essential oil (MEO) had the most significant inhibitory effect on *Aspergillus flavus* [9,10]. The EOs of lemon, mandarin, grapefruit and orange has shown inhibitory effects on the growth of some bacteria in the food industry, (*Lactobacillus curvatus*, *L. sakei*, *Staphylococcus carnosus* and *S. xylosus*) and those related to food spoilage (*Enterobacter gergoviae* and *E. amnigenus*) in the food industry [11]. With the exception of some phototoxicity of expressed oils, they are generally safe to use with negligible toxicity to humans. These readily available essential oils will undoubtedly continue to play important roles in the food and beverage industries [7].

The high incidence of food poisoning and foodborne diseases originating from microbial spoilage during processing and storage is a serious threat to public health and has generated wide global concern in recent years. *Staphylococcus aureus (S. aureus*) is a common pathogenic bacterium that causes food poisoning and purulent diseases, is extensively distributed in water, air, dust and animal faces [11]. *S. aureus* is highly resistant to environmental pressures and attaches easily to the surface of the food processing instruments, thereby polluting products such as milk, meat, eggs and fish [12]. Little is known about the mechanism by which MEO inhibits *S. aureus*; however, research into the action of MEO on *S. aureus* is seen to be significant for enhancing the broad application of EOs in different industrial fields. Therefore, the objective of this study was to investigate the antibacterial activity of MEO against *S. aureus*, and to study the antibacterial mechanism on bacterial cell membrane would be further clarified.

## 2. Results and Discussion

### 2.1. Analysis of Chemical Compounds in MEO

The GC-MS analysis of the chemical compounds in MEO is presented in Table 1. A total of seven different compounds were identified. As the principal compound in MEO, (−)-limonene displayed the highest content (78.89%), followed by γ-terpinene (14.56%). Other compounds identified included α-pinene (2.09%), β-pinene (1.51%), β-myrcene (1.24%), *o*-cymene (1.11%) and b-thujene (0.7%) These GC-MS results are consistent with previous reports [13]. Limonene, is thus considered to be the main constituent involved in the exertion of MEO’s bioactivity against bacteria and fungi [14,15].

### 2.2. Antibacterial Effect of MEO on Planktonic S. aureus

The MIC value was found to be 0.5 mg/mL and the 2MIC value was 1 mg/mL. Previous studies showed that there are qualitative similarities between components present in oils although some of them showed quantitative differences when tested. The components are grouped into several classes: hydrocarbon monoterpenes, oxygenated monoterpenes, hydrocarbon sesquiterpenes, oxygenated sesquiterpenes and others [16]. The antimicrobial effects of the essential oils of three commercially grown citrus fruits (orange, lemon and mandarin) against food spoilage caused by pathogenic microorganisms were reported, and found the mandarin essential oil to possess the broadest spectrum of actions [14]. Oxygenated monoterpenes in essential oils extracted from the fruit peel of citrus genotypes (orange, mandarin and lemon) were most effective in inhibiting the growth of *Listeria* spp. strains and played a direct role in their antibacterial mechanisms [17]. In general, researchers tend to report that the antibacterial effects of MEOs are related to the compounds and the species or isolates under study [18]. In this study, it was shown that the MEO had a prominent inhibitory effect on *S. aureus* growth. Furthermore, the antibacterial mechanism involved in the MEO’s action against planktonic *S. aureus* was also investigated.

### 2.3. Antibacterial Mechanism of MEO Against Planktonic S. aureus

#### 2.3.1. Effect of MEO on *S. aureus* Cell Morphology

**Figure 1:**
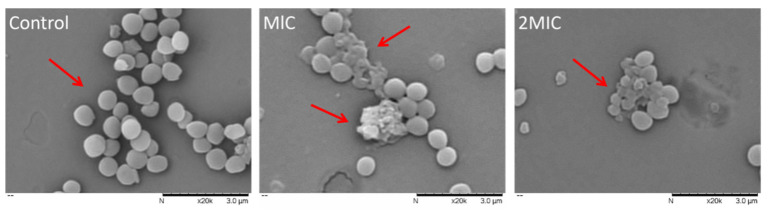
Scanning electron micrographs of *S. aureus* (untreated, treated with MEO at MIC and 2MIC, respectively).

Figure 1 displays the morphological changes of both the treated and untreated *S. aureus*. It shows that the control cells of *S. aureus* had a normal shape, with an intact membrane, retained normal cell morphology and inner structure, but that damage (indicated by red arrows) had formed on the surface of cells related with MEO at MIC and 2MIC; the obvious distortion of *S. aureus* cells, with an unclear profile, collapsed surface and pitted, damaged cell membrane and membrane disruption, was also observed. Moreover, the cells treated with MEO at 2MIC concentration were found to be severely damaged and the original integrity of the cell membrane completely disappeared with leakage of intracellular substances. *Cyperus rotundus* rhizomes essential oil possibly caused the loss of *S. aureus* cell viability by directly changing the integrity of cell membranes and cell walls [19]. Similarly, our previous study [20] indicated that the destruction of *S. aureus* cell membrane by peppermint essential oil was irreversible. The influences of antibacterial agents on the morphology of bacterial cells were varied, such as separation of cytoplasmic membrane from cell wall, cytoplasmic content leakage, cell lysis and cell distortion [21,22]. Our results showed that MEO could cause the destruction of cell membranes integrity and leakage of intracellular substances. We further examined the cell membrane permeability and integrity.

#### 2.3.2. Effect of MEO on *S. aureus* Cell Membrane Potential

**Figure 2:**
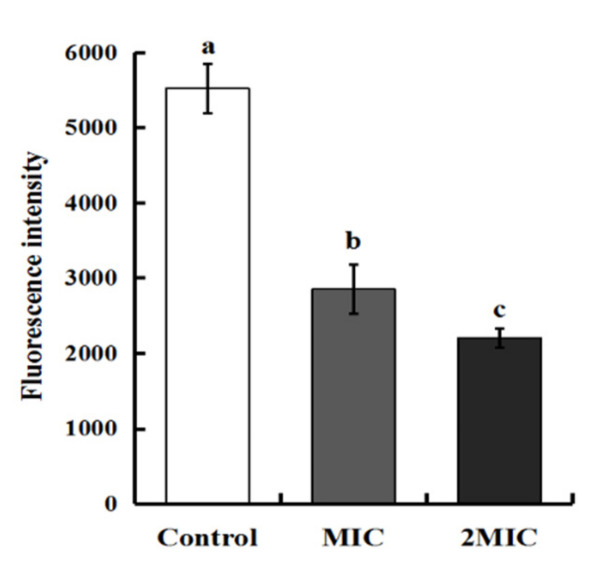
Effect of MEO on membrane potential of *S. aureus.* Difference letters means statistical difference.

Rhodamine 123 was used to detect changes in the potential of the *S. aureus* cell membrane. As shown in Figure 2, the fluorescence intensity of *S. aureus* cells treated with MIC level was significantly reduced by 48.31%. With MEO concentration increased to 2MIC, a greater fluorescence intensity decrease of 60.05% was observed in *S. aureus*. These results demonstrate that the cell membrane potential was obviously reduced and depolarized by the effects of MEO in a dose-dependent manner. Membrane potential is defined as the potential difference between the interior and exterior of a biological cell. Normal mitochondrial membrane potential can, for example, maintain mitochondrial oxidative phosphorylation levels and ATP production [23]. The results of this study found that the depolarization of cells exposed to MEO could result in abnormal metabolic activities of the cells, and the SEM images also displayed the structural distortion and serious destruction of *S. aureus* cell treated with MEO. The *Listeria monocytogenes* cells was treated with tannin-rich fractions and found that the cell membrane potential was significantly changed and depolarized [23]. Similar findings were reported when *S. aureus* was exposed to melanin from Lachnum YM30 [24].

#### 2.3.3. Effect of MEO on *S. aureus* Cell Constituents

**Figure 3:**
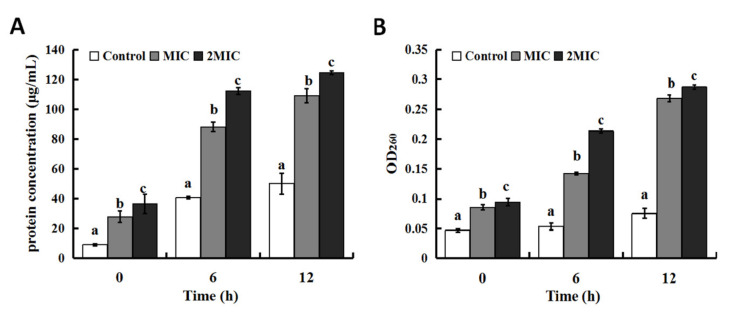
Effect of the MEO on the release of *S. aureus* cells constituents. (**A**) Leakage of protein from the membrane of *S. aureus* treated with MEO; (**B**) Release of intracellular nucleic acids from *S. aureus* treated with MEO. Difference letters means statistical difference.

The protein and the nucleic acid leakage through the membrane in the *S. aureus* cells exposed to MEO at MIC and 2MIC is shown in Figure 3. After MEO treatment at MIC level, the extracellular protein quantity of the *S. aureus* cells was found to have increased by 3.06, 2.17 and 2.18 times at 0, 6 and 12 h, respectively. When the *S. aureus* cells were treated at the 2MIC level, extracellular protein quantity increased by 3.99, 2.76 and 2.49 times at 0, 6 and 12 h, respectively. Further analysis of the nucleic acid quantity of the cell-free supernatants revealed that the absorbance values of the nucleic acids of *S. aureus* cells were correspondingly enhanced following increases in the concentrations of MEO (MIC and 2MIC). The nucleic acid exhibited a progressive release within 12 h, and nucleic acid release rate at 2MIC level was faster compared to that at the MIC level. The destruction of the cell membrane, a pivotal structural component of a bacteria cell, will lead to the leakage of nucleic acids, protein and other intracellular substances. Therefore, the leakage of intracellular constituents can be used as a typical indicator to determine the integrity of the cell membrane [23]. It has been found that cinnamon EO could affect the integrity of a cell membrane by acting on it and, thereby, inducing the loss of cell membrane function and the leakage of nucleic acids and proteins [25]. Furthermore, in our previous study it was found that that peppermint EO caused the destruction of the *S. aureus* cell membrane, which raised the level of extracellular nucleic acids and proteins [20]. Similarly, these current results suggest that the MEO’s irreversible damage to the *S. aureus* cell membrane integrity led to the leakage of cell constituents and cell death.

#### 2.3.4. Effect of MEO on *S. aureus* Cell Proteins

**Figure 4:**
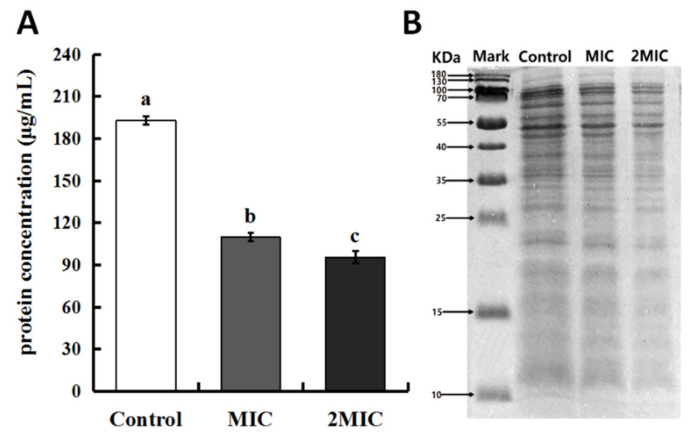
The effect of MEO on the protein of *S. aureus.* (**A**) The effect of MEO on the intracellular protein of *S. aureus*; (**B**) SDS-PAGE profile of intracellular proteins of *S. aureus* cells treated with MEO. Difference letters means statistical difference.

Changes in the quantity of intracellular and whole-cell proteins were further evaluated. As shown in Figure 4, it was found that the intracellular protein quantities in the *S. aureus* cells were significantly lower compared to that of the control, and the levels of protein loss were more extensive as the concentrations of MEO increased. The results of electrophoresis also reflected the loss of proteins. The SDS-PAGE pattern showed that the protein bands of the control were clear, while the protein bands of the samples gradually became shallow or even disappeared after 12 h of treatment with MEO. These combined results suggest that the MEO treatment affected the protein synthesis of *S. aureus* cells. Proteins are essential biological macromolecules in the cell membrane and cytoplasm of bacteria [26,27,28]. In previous studies, we used SDS-PAGE to analyze the total soluble proteins of *Bacillus cereus* exposed to thyme EO (TEO) and observed that TEO could either inhibit the release of protein or destroy cell protein synthesis to inhibit bacterial activity [29]. This is similar to the whole-cell protein loss observed in this study in response to MEO treatment. The results confirmed that TEO could not only damage the integrity of cell membranes, leading to the leakage of intracellular constituents, but could also possibly inhibit the synthesis of proteins to kill bacterial cells. It has been established that clove oil could affect the synthesis of nucleic acid and the expression of related genes of *L. monocytogenes* to hinder the synthesis of intracellular proteins and enzymes [30].

#### 2.3.5. Effect of MEO on Intracellular ATP Concentrations of *S. aureus*

**Figure 5:**
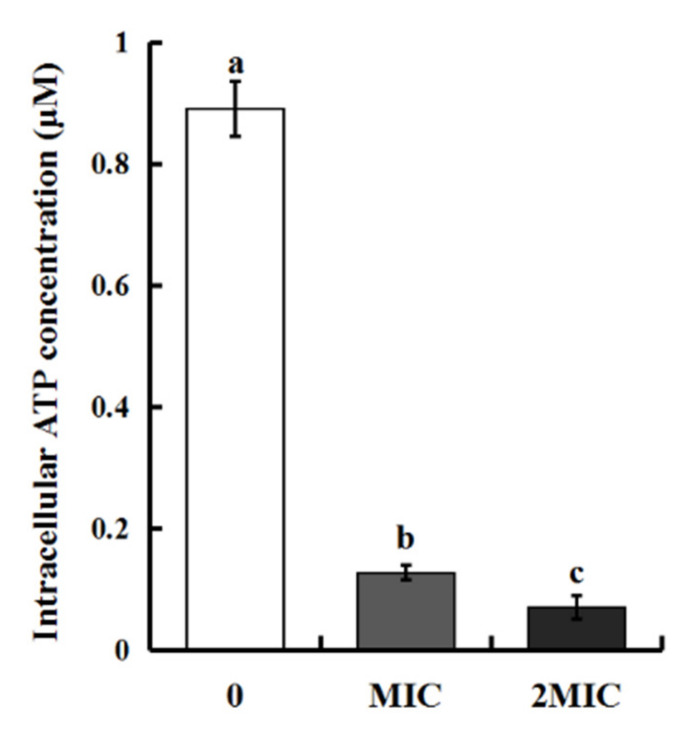
Effect of MEO on the intracellular ATP concentration of *S. aureus.* Difference letters means statistical difference.

The intracellular ATP level of *S. aureus* was determined, and the ATP concentration was significantly reduced after the treatment with MEO, as shown in Figure 5. The intracellular ATP concentration of *S. aureus* in the untreated control was 0.89 μM, while there were obvious decrease by 85.58% and 92.02% after treatment with MEO at MIC and 2MIC, compared to the levels in the untreated cells. ATP is essential for bacterial cell functions, including energy conversion requirements, nutrient processing and transportation, the synthesis of structural macromolecules and the secretion of various enzymes [31] and could, thus, be used to understand the antibacterial mode of MEO. The results suggest that reduced intracellular ATP concentration could inhibit ATPase activity and ATP synthesis. Previous studies treated *Escherichia coli* and *S. aureus* with cinnamon EO and found that intracellular ATP concentration sharply decreased while extracellular ATP concentration increased, suggesting that cinnamon EO could enhance the permeability of cell membranes and inhibit the synthesis of ATP [32].

## 3. Materials and Methods

### 3.1. Materials and Culture

The MEO was kindly provided by the Oshadhi Co., Ltd. (Baden-Baden, Germany). The strain of *Staphylococcus aureus* (*S. aureus*, ATCC 25923) was supplied by the Food Safety and Hygiene Laboratory (Shaanxi Normal University, Xi’an, China), and stored in cryovials (containing Luria-Bertani (LB) medium with 25% (*v*/*v*) glycerol) at −20 °C. Before each experiment, the strains were reactivated and shake-cultured into LB broth overnight at 37 °C. All other chemicals were of analyzed grade and provided by JingBo Biotechnology Co., Ltd. (Xi’an, China).

### 3.2. Chemical Compounds for the MEO Analysis

The MEO was diluted with hexane to 1% (*v*/*v*) and analyzed by means of a QP-2010 Ultra system (Shimadzu, Tokyo, Japan). The gas chromatograph-mass spectrometry (GC-MS) analysis was performed as previously described [19]. An HP-5MS capillary column (30 m × 250 µm, film thickness 0.25 µm) was used for the separations. The temperature program was maintained at 50 °C for 1 min, increased to 250 °C at a rate of 5 °C /min, and maintained for a further 5 min. The column pressure was 50 kPa, and the carrier gas (helium, 1.2 mL/min) was at a split ratio of 1:50. The MS was operated in electron impact mode. The ionization energy was 70 eV. The ion source temperature was 230 °C, scanning mass range of 20–500 amu.

The identification of MEO chemical compounds were done using GC-MS software (GC-MS Postrun Analysis) and computer matched within the NIST14 library. The relative percentage of components in the MEO calculated by peak area normalization.

### 3.3. Determination of Minimal Inhibitory Concentration (MIC) 

The determination of MIC values of the MEO against the *S. aureus* were determined by the double broth dilution method which was modified according to a previously described method [20]. MEO (dissolved in dimethyl sulfoxide (DMSO), 0.5% (*v*/*v*)) was added to the sterile LB broth to a concentration of 16 mg/mL, and then two-fold serially diluted with LB broth in 10 mL test tubes to the final concentration ranges from 0.25 to 16 mg/mL (0.25, 0.5, 1, 2, 4, 8, 16 mg/mL). Finally, 50 μL of *S. aureus* suspension (10^7^ CFU/mL) was inoculated into each test tube and all tubes were then shake-incubated (160× *g*) at 37 °C for 24 h. The MIC values were defined as the lowest concentration of MEO inhibiting *S. aureus* growth during the incubation period. 

### 3.4. Antibacterial Mechanism of MEO Against S. aureus

#### 3.4.1. Scanning Electron Microscopy (SEM) Analysis

The *S. aureus* were treated based on the method provided by previous study [33]. *S. aureus* suspension (10^7^ CFU/mL) was either treated or untreated with MEO at MIC and 2MIC concentrations for 6 h at 37 °C. Thereafter, the suspensions were washed with PBS and centrifuged at 5000× *g* for 10 min at 4 °C, and precipitated cells were fixed in 2.5% glutaraldehyde at 4 °C for 6 h. Subsequently, the fixed cells were dehydrated with a series of different concentrations (25%, 50%, 75%, 95%, and 100%) of ethanol for 10 min. Finally, the dehydrated samples were coated with gold, and observed by a SEM (TM3030, Hitachi, Tokyo, Japan).

#### 3.4.2. Determination of *S. aureus* Cell Membrane Potentials

The potentials of *S. aureus* cell membrane treated with MEO at various concentrations (MIC and 2MIC) were determined according to the reported procedures with slight modifications [23,29]. The bacterial suspension (10^7^ CFU/mL) was separated and treated with MEO (MIC and 2MIC) for 4 h. Then, the treated or untreated *S. aureus* suspensions were washed and re-suspended in PBS. Rhodamine 123 (Sigma-Aldrich, St. Louis, MO, USA) was added to each sample and mixed to a final concentration of 5 μg/mL. Finally, all samples were incubated at 37 °C for 30 min in darkness, then centrifuged and resuspended in PBS. The fluorescence values of samples were immediately measured using a fluorescence microplate reader (RT-6000, Biotek, Winooski, VT, USA). Excitation and emission wavelengths were recorded at 480 and 530 nm, respectively.

#### 3.4.3. Determination of Extracellular Nucleic Acid and Protein Concentrations

The leakage cell constituents were ascertained by determining extracellular nucleic acid and proteins, according to a previously described method [20,29]. *S. aureus* suspensions were exposed to different concentrations of MEO (0 (control), MIC and 2MIC) of MEO for 0, 6, and 12 h, the sample untreated with MEO was regarded as the control. Thereafter, the cells suspensions were centrifuged for 10 min at 5000× *g*, and the supernatants were collected to measure the extracellular nucleic acid using a UV-visible spectrophotometer (Beijing Persee General Instrument Co., Ltd., Beijing, China). The quantity of protein leakage through the cell membrane was detected using a BCA protein assay kit (Sangon, Shanghai, China).

#### 3.4.4. Determination of Intracellular Protein Concentrations

The treatment of *S. aureus* cells was established as described in Section 3.4.3 except that the precipitated cells exposed to MEO for 12 h were tested for protein quantity with a micro BCA protein assay kit (Sangon, Shanghai, China).

#### 3.4.5. Determination of *S. aureus* Cell Whole-Cell Proteins

Sodium dodecyl sulfate-polyacrylamide gel electrophoresis (SDS-PAGE) was performed to analyze the cellular proteins according to a previously described method [33]. The collected samples were consistent with those described in Section 3.4.4. The samples were mixed with 5X protein loading buffer, whereafter the mixtures were boiled for 5 min, then cooled and centrifuged. The supernatants of the *S. aureus* cells were collected for SDS-PAGE analysis. An SDS-PAGE gel preparation kit (Solarbio, Beijing, China) was used to prepare the gel (5% stacking gel and 12% separating gel). After electrophoresis, the gel was stained with Coomassie brilliant blue R-250 and decolorized for analysis.

#### 3.4.6. Determination of Intracellular Adenosine Triphosphate (ATP) Concentrations

The intracellular ATP concentrations of *S. aureus* cells treated or untreated with MEO at MIC and 2MIC concentrations, was determined according to a previously described method [24], with some modifications. Briefly, *S. aureus* suspensions (10^7^ CFU/mL) were removed to 4 mL centrifuge tube with LB broth and exposed to MEO at 0 (control), MIC or 2MIC, and then incubated at 37 °C for 30 min. The samples were then centrifuged at 5000× *g* for 5 min and the precipitated cells were collected to determine intracellular ATP concentrations. Furthermore, all samples were treated with ultrasound during the extraction of the intracellular ATP, which were kept on ice to prevent loss. The intracellular ATP concentrations of *S. aureus* cells were measured using an ATP assay kit (Beyotime Bioengineering Institute, Shanghai, China) according to the manufacturer’s instructions.

### 3.5. Statistical Analysis

All experiments were performed in triplicate. The data were analyzed using IBM SPSS software (version 21.0) (IBM Corp., Armonk, NY, USA). One-way analysis of variance and the least significance test were used to detect differences at *p* < 0.05.

## 4. Conclusions

In conclusion, MEO had potent antibacterial against *S. aureus*. The cell membranes of *S. aureus* exhibited irreversible damage after the MEO treatment, and this disruptive effect was confirmed by increased cell membrane permeability, increased leakage of nucleic acids, proteins and ATP, and changes in bacterial morphology. The findings of this study confirm that MEO can penetrate the bacterial membrane and act on the cell membrane to kill *S. aureus* cells, thus establishing its potential as a representative natural antimicrobial agent for food preservation.

## Figures and Tables

**Table 1:** Chemical composition (%) of MEO.

Peak No.	RT (min)	Compounds	CAS No.	Percentage (%)
1	5.121	b-thujene	28634-89-1	0.7
2	5.287	α-pinene	80-56-8	2.09
3	6.336	β-pinene	127-91-3	1.51
4	6.636	β-myrcene	123-35-3	1.24
5	7.57	*o*-cymene	527-84-4	1.11
6	7.685	(−)-limonene	5989-54-8	78.79
7	8.485	γ-terpinene	99-85-4	14.56

RT: retention time.
